# The Duodenal Accessory Ampulloma and the Role of Endoscopic Retrograde Cholangiopancreatography (ERCP) in Familial Adenomatous Polyposis Coli Inheritance

**DOI:** 10.7759/cureus.59445

**Published:** 2024-05-01

**Authors:** Muhammad Ibrahim Shahzad, Grace Pickering, Mansoor Zafar, Sulaiman Hayat, Panagiotis Vlavianos

**Affiliations:** 1 Gastroenterology, Hammersmith Hospital, Imperial College Healthcare NHS Trust, London, GBR; 2 Gastroenterology, Imperial College London, London, GBR; 3 Medicine, Hammersmith Hospital, Imperial College Healthcare NHS Trust, London, GBR; 4 Hepatobiliary and Gastroenterology, Hammersmith Hospital, Imperial College Healthcare NHS Trust, London, GBR

**Keywords:** familial adenomatous polyposis, apc gene mutation, apc gene, duodenal accessory ampulloma, duodenal ampulloma

## Abstract

The adenomatous lesions, which could be benign or malignant, have been described in the duodenum and along the duodenal ampulla in individuals with familial adenomatous polyposis (FAP) post-colectomy, along with other extracolonic manifestations. To our best knowledge, we present a unique case of the involvement of the accessory duodenal ampulla in a patient who had undergone colectomy with ileorectal anastomosis with an established diagnosis of FAP. During the endoscopic examination, the patient was found to have adenomatous growth in the accessory duodenal ampulla, which was successfully removed via endoscopic retrograde cholangiopancreatography (ERCP). To prevent pancreatitis, a temporary plastic stent was inserted and successfully removed three weeks later.

## Introduction

The prevalence of familial adenomatous polyposis (FAP) globally is estimated at three cases per 100,000 people [1]. FAP is an inherited autosomal dominant condition and stems from a mutation of the *adenomatous polyposis coli* (*APC*) gene on the long arm of chromosome 5 [2,3]. It has been associated with the early development of significant numbers of adenomas throughout the colon. Colorectal cancer develops in virtually all affected members by the fifth decade of life unless prophylactic colectomy is performed [4].

The extra-colonic intestinal manifestations include polyposis in the fundic glands of the stomach, adenomas in the antrum of the stomach, and adenomas in the small intestine. The risk for recurrence of adenomas in various segments of the duodenum, adenomas in the duodenal ampulla, and associated malignancies in the upper gastrointestinal tract has been reported to be increased post-colectomy with a remnant or retained rectum following ileorectal anastomosis, as observed in our patient [5-8].

## Case presentation

A 30-year-old male was referred from the district general hospital to the tertiary centre due to uncertainty over a duodenal ampullary lesion found during an annual surveillance upper gastrointestinal endoscopy.

He had a history of FAP with a pathogenic germline variant of the *APC* tumour suppressor gene located on chromosome 5 (5q21‐22; OMIMNM_000038.5), and his genetic analysis in the past showed a pathogenic p.Arg213Ter (c.637C>T) variant in exon 5 at codon 213 of the *APC* gene. His family history included similar genetic predispositions found in his mother and maternal grandmother.

He previously had a yearly surveillance colonoscopy, and during each procedure, an average of 20-80 polyps were found and removed. Hence, he agreed to undergo an elective laparoscopic colectomy followed by ileorectal anastomosis 14 years ago to address the presence of significant polyps throughout the large bowel. His long-term management plan consists of undergoing colonoscopies every six months and upper gastrointestinal endoscopies yearly for surveillance purposes.

**Figure 1:**
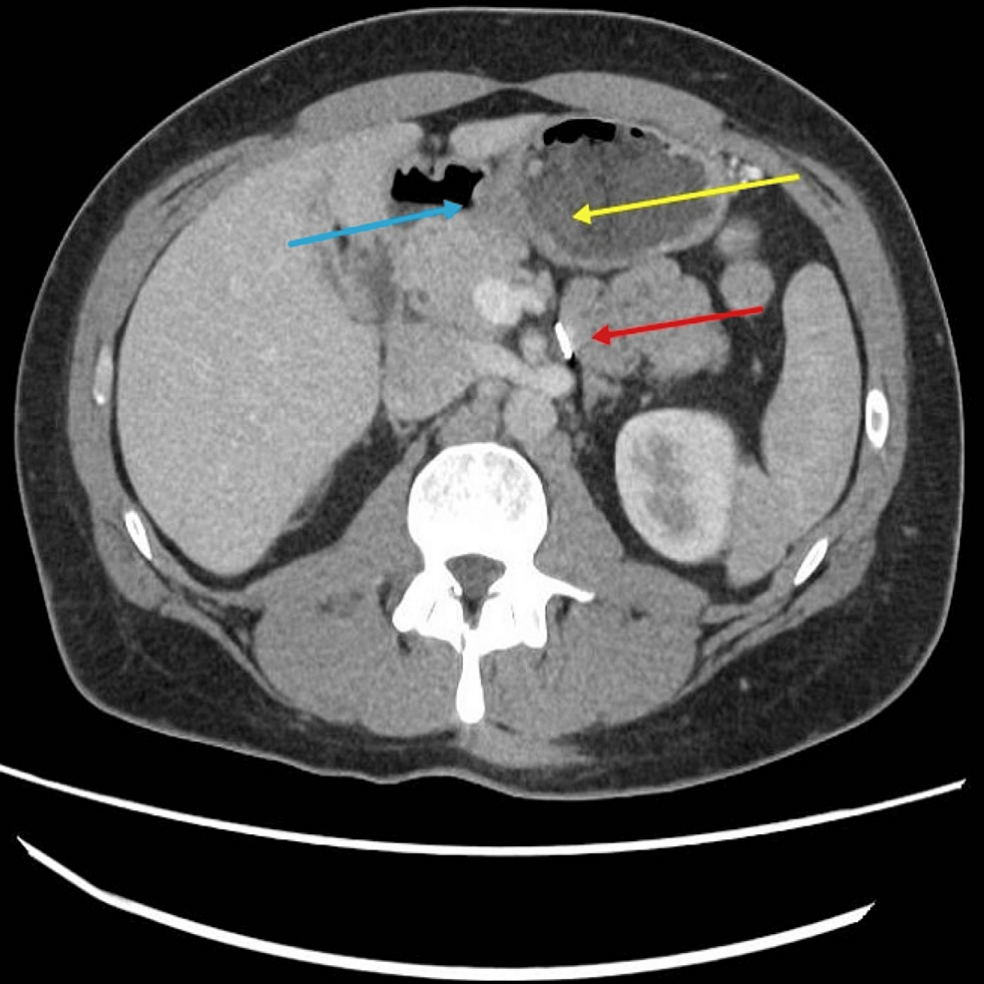
Axial view of the abdomen-pelvis computed tomogram (CT) scan showing a fluid-filled duodenum (yellow arrow) with a query lesion along the D2 (blue arrow). Additionally, surgical clips from a previously elected laparoscopic colectomy are observed (red arrow).

At the tertiary centre, he was clinically asymptomatic, and his routine blood tests were all within the normal reference range. He underwent a computed tomogram (CT) of the abdomen-pelvis that showed a fluid-filled duodenum and a focal ill-defined 2.5 cm diameter thickening of the medial wall at the ampulla. There was no dilatation of the biliary or pancreatic ducts, and the pancreas exhibited normal enhancement (Figure 1).

**Figure 2:**
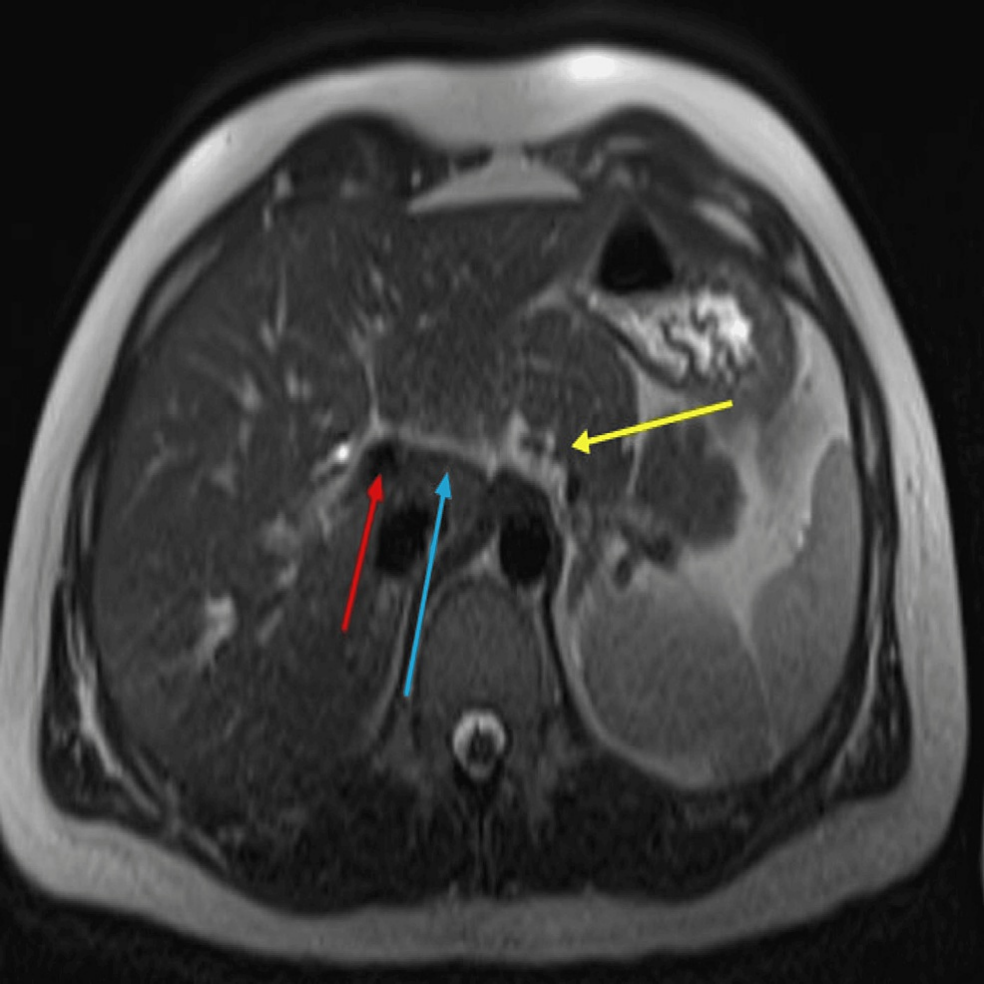
The magnetic resonance cholangiopancreatography (MRCP) image showing a collapsed duodenum with a focal thickening of the medial wall of the D2 (yellow arrow). Additionally, the dominant dorsal pancreatic duct (blue arrow) and biliary duct (red arrow) are also visible.

Subsequently, he had a magnetic resonance cholangiopancreatogram (MRCP) that showed a collapsed duodenum. No focal lesion could be clearly identified except for an indeterminate focal area of thickening of the medial wall of the second segment of the duodenum (D2). No focal abnormality or significant upper abdominal lymphadenopathy was noticed. The gallbladder appeared normal, with no bile duct or pancreatic duct dilatation. However, a dominant dorsal pancreatic duct was observed (Figure 2).

**Figure 3:**
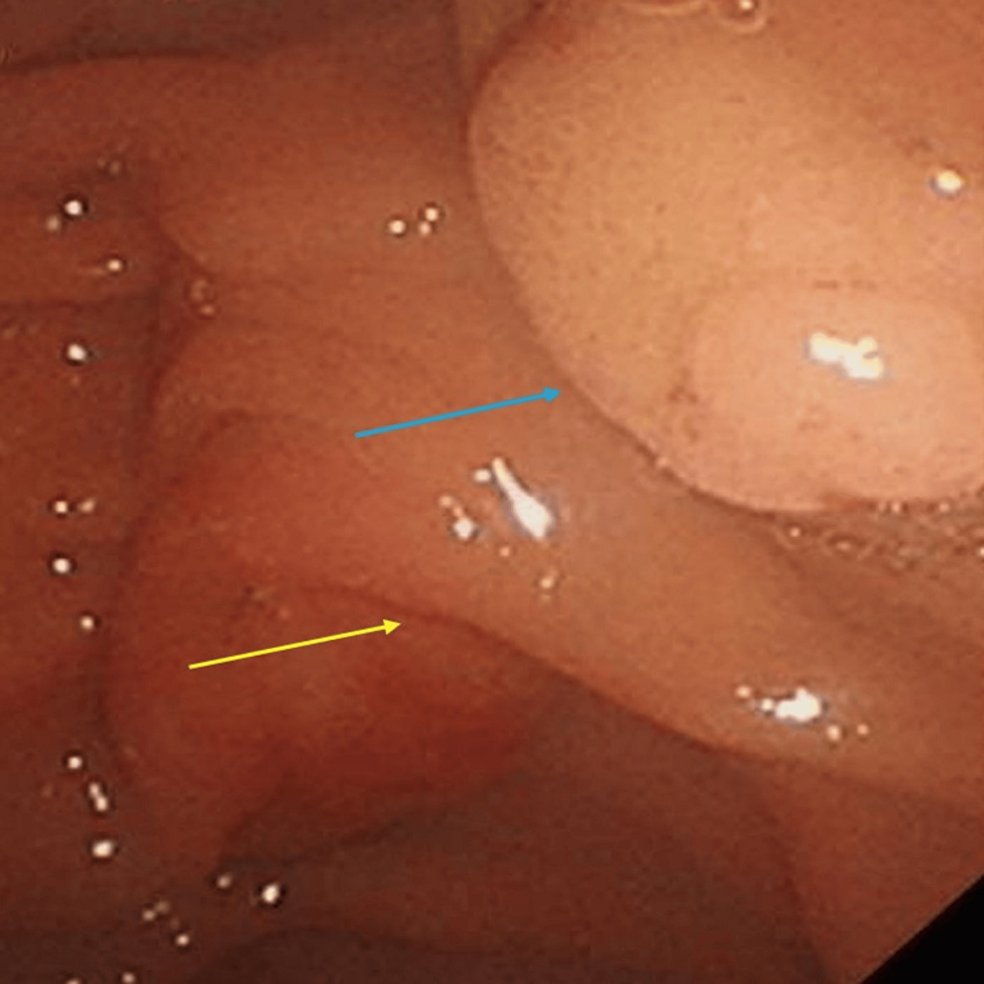
Upper gastrointestinal endoscopy confirming duodenal ampullary ampulloma at the deeper minor papilla (blue arrow) surrounded by the major papilla superficially (yellow arrow).

The patient underwent an upper gastrointestinal endoscopy that confirmed the ampulla lesion along the accessory duodenal ampulla, which appeared to be resectable endoscopically (Figure 3).

**Figure 4:**
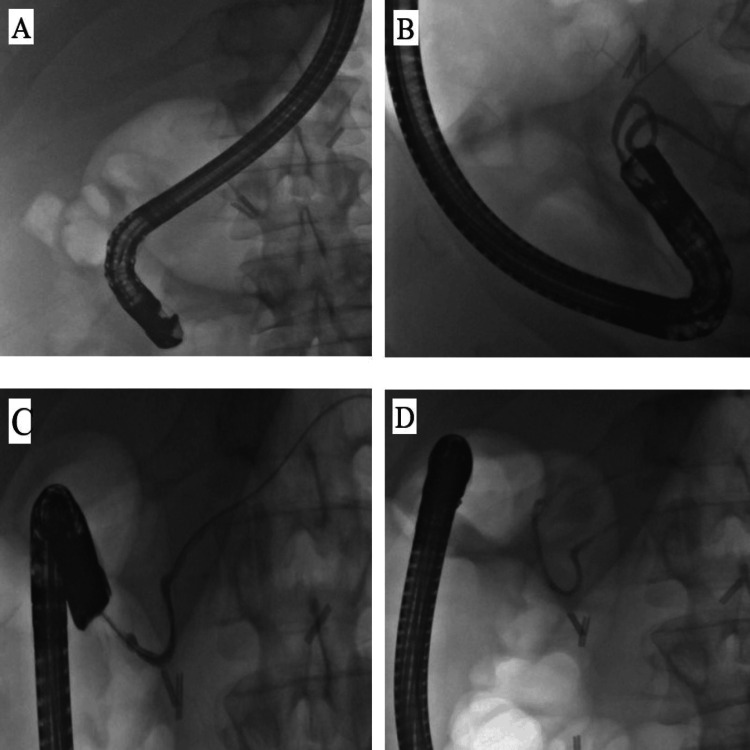
Successful cannulation of the ampulla (A), followed by cannulation of the dorsal pancreatic duct (B) confirmed with a minor contrast injection (C) and the insertion of a 5Frx 7 cm single pigtail stent to prevent pancreatitis (D).

During ERCP, the scope progressed in a long position to the D2. The major papilla was visualised, which did not show any adenomatous lesion. The minor papilla was seen just 1 cm above the major papilla and showed an adenoma of 8 mm diameter and no endoscopic signs of deep invasion. Complete resection of this ampulloma was performed using a 10 mm snare, and the ampulloma was retrieved for histology. The cannulation of the dorsal pancreatic duct was confirmed with a minor contrast injection, and a 5Frx 7 cm single pigtail stent was inserted to prevent pancreatitis (Figure 4).

The patient was admitted to the hepatobiliary ward and prescribed nil by mouth (NBM) for four hours, then a normal diet once pain-free. The patient was discharged the next day as he was pain-free and tolerating oral feeding satisfactorily, with a request for a plain abdominal X-ray as an outpatient in two weeks to confirm stent migration. The patient was informed that in the event of non-migration of the stent, a repeat scoping would be required to remove the plastic stent. The abdominal X-ray two weeks post-ERCP confirmed that the plastic stent was not migrating. The patient was rescoped three weeks post-ERCP, and the plastic stent was removed with no complications. The biopsy report revealed that the ampulloma showed tubular adenoma with no dysplasia and no serious consequences. Hence, the patient will be discharged back to the community with a follow-up duodenoscopy of the resection site in three months. Following that, he would need to continue with the surveillance regime of biannual colonoscopies and yearly oesophago-gastro-duodenoscopy (OGD) at the local district general hospital.

## Discussion

In 1904, the discovery of duodenal polyps was documented [6], and to this day, the duodenum continues to be a prevalent site for polyps, second only to the colon [7-9]. Duodenal adenomas have been reported in 30%-70% of FAP patients, and the lifetime risk of duodenal adenomas in FAP patients approaches 100% [10].

Duodenal and periampullary adenocarcinomas are the leading cause of death in patients with FAP after colorectal cancer [11]. Additionally, it has been reported that there is a 100-330-fold increased risk of duodenal cancer in FAP patients when compared with the general population [12].

Prophylactic colectomy remains the most important management for patients diagnosed with FAP, as there is a 100% risk of colorectal malignancy [13]. However, most patients who undergo prophylactic colectomy and proctocolectomy have been noted to develop extracolonic manifestations (ECM), including malignancies with increased mortality [14]. The list is extensive but includes sebaceous cysts, epidermoid cysts, lipomas, fibromas, desmoid tumours, dental abnormalities, congenital hypertrophy of retinal pigment epithelium lesions (CHRPE), osteomas, and gastric and duodenal polyps [13]. The malignancies reported include those affecting the brain, thyroid, stomach, duodenum, biliary tree, pancreas, and hepatoblastoma [15]. The most common lesions reported in the upper gastrointestinal tract in post-colectomy patients with a diagnosis of FAP include fundic gland polyps, gastric adenomas, duodenal adenomas, and carcinomas [10].

The majority of adenomas in various segments of the duodenum and along the ampulla are ideally managed with the removal of polyps, commonly regarded as polypectomy and ampullectomy when an adenoma involves the ampulla. The duodenal adenomas are also managed by direct destruction via ablation techniques, namely, thermal ablation, argon plasma coagulation, or photodynamic therapy [15,16]. Although duodenal polypectomy and ampullectomy are low-risk procedures, they have still been reported to be associated with consequences including bleeding, perforation, and pancreatitis [17].

During an ampullectomy, it is important to insert a plastic stent into the pancreatic duct post-procedure to avoid the associated risk of pancreatitis [16,17]. This cannulation of the pancreatic duct requires inserting a guide wire through the sphincter of Oddi at the pancreatic duct at the major duodenal papilla, also known as the ampulla of Vater, which encompasses and is connected to the common bile duct and the duct of Wirsung (the main pancreatic duct) and runs relatively ventrally. Pancreatic divisum is an anomaly wherein the dorsal and ventral ductal systems fail to fuse. Hence, the majority of pancreatic secretions are drained through the accessory pancreatic duct, also known as the duct of Santori (accessory pancreatic duct), that runs dorsally. There are variations of the pancreatic divisum, and hence, the common bile duct (CBD) may open independently into the duodenum with the main pancreatic duct. However, the accessory pancreatic duct is functional, or it may empty into the duodenum along with the main pancreatic duct, and the minor duodenal ampulla ends blindly. When the accessory duct is responsible for the drainage of the majority of the pancreas instead of the main pancreatic duct, the phenomenon is called pancreatic divisum, and it occurs in 10% of the population [18].

The surveillance of post-colectomy FAP patients remains controversial. Some reports suggest that surveillance should begin when FAP is diagnosed, whilst other reports propose not to initiate surveillance until the patients reach the age of 25-30 years, as duodenal cancer diagnosis before age 30 remains a rarity [15,19]. The recommendations for post-baseline evaluation remain according to the Spigelman stage of disease severity [7].

Lastly, the finding of an accessory ampulla leading to an accessory pancreatic duct that ran dorsally and needed cannulation to insert a temporary plastic stent in our patient to prevent pancreatitis is unique. Although the literature commonly mentions excision of ampulloma along the major duodenal ampulla, the presence of accessory ampulloma that required excision is a unique outcome that, to the best of our knowledge, we have reported for the first time. It will be interesting to see more case reports, case series, and research to further expand on the understanding of the complexities of caring for patients with FAP, who post-colectomy remain at high risk of recurrence of adenomatous polyps or occurrence of malignancies in various segments of the duodenum, duodenal ampulla, and now accessory ampulla.

## Conclusions

The association of duodenal ampullary papilloma is a known phenomenon managed with ampullectomy. However, to the best of our knowledge, this is the first case of a duodenal ampullary papilloma being found on the accessory duodenal ampulla, signifying the importance of understanding that the papilloma could also be found on the accessory ampulla, which, on imaging modalities, may only show as focal thickening along the medial wall of the second segment of the duodenum. This suggests the importance of direct visualisation via ERCP to optimise care of papillectomy, as they could be benign as well as malignant.

This also signifies the importance of periodic upper GI endoscopy surveillance in such patients every year and colonoscopy surveillance on a six-month basis, as the risk of cancer exists in such patients despite prior ileocolonic resection.
